# Performance of a Point-of-Care Test for the Rapid Detection of SARS-CoV-2 Antigen

**DOI:** 10.3390/microorganisms9010058

**Published:** 2020-12-28

**Authors:** Annabelle Strömer, Ruben Rose, Miriam Schäfer, Frieda Schön, Anna Vollersen, Thomas Lorentz, Helmut Fickenscher, Andi Krumbholz

**Affiliations:** 1 Institute for Infection Medicine, Christian-Albrecht University and University Medical Center Schleswig-Holstein, Brunswiker Str. 4, 24105 Kiel, Germany; annabelle.stroemer@gmail.com (A.S.); rose@infmed.uni-kiel.de (R.R.); fickenscher@infmed.uni-kiel.de (H.F.); 2 Labor Dr. Krause und Kollegen MVZ GmbH, Steenbeker Weg 23, 24106 Kiel, Germany; schaefer@labor-krause.de (M.S.); schoen@labor-krause.de (F.S.); anna.vollersen@hotmail.com (A.V.); lorentz@labor-krause.de (T.L.)

**Keywords:** COVID-19, diagnostic, PCR, antigen, POCT, comparison

## Abstract

The rapid detection of infections caused by the *severe acute respiratory syndrome coronavirus 2* (SARS-CoV-2) is necessary in the ongoing pandemic. Antigen-specific point-of-care tests (POCT) may be useful for this purpose. Here, such a POCT (SARS-CoV-2 NADAL^®^ COVID-19 Ag) was compared to a laboratory-developed triplex real-time polymerase chain reaction (RT-PCR) designed for the detection of viral nucleoprotein gene and two control targets. This RT-PCR served as a reference to investigate POCT sensitivity by re-testing upper respiratory tract (URT) samples (*n* = 124) exhibiting different SARS-CoV-2 loads in terms of RT-PCR threshold cycle (Ct) values. The optical intensities of the antigen bands were compared to the Ct values of the RT-PCR. The infectivity of various virus loads was estimated by inoculating Vero cells with URT samples (*n* = 64, Ct 17-34). POCT sensitivity varied from 100% (Ct < 25) to 73.1% (Ct ≤ 30); higher SARS-CoV-2 loads correlated with higher band intensities. All samples with a Ct > 30 were negative; among SARS-CoV-2 free samples (*n* = 10) no false-positives were detected. A head-to-head comparison with another POCT (Abbott, Panbio™ COVID-19 Ag Rapid Test) yielded similar results. Isolation of SARS-CoV-2 in cell-culture was successful up to a Ct value of 29. The POCT reliably detects high SARS-CoV-2 loads and rapidly identifies infectious individuals.

## 1. Introduction

At the end of 2019, local health authorities reported unusual cases of pneumonia in Wuhan, a large city in the Hubei Province, China [1]. Shortly afterwards, a novel beta-coronavirus has been identified as the causative agent of the disease. This virus emerged globally and is designated as *severe acute respiratory syndrome coronavirus 2* (SARS-CoV-2) [1,2,3]. As of 25 November 2020, the World Health Organization reported 59,204,902 SARS-CoV-2 infections (COVID-19) and 1,397,139 deaths worldwide. Only a few days after the first report of this novel airway infection, Corman et al. published three one-step reverse transcription real-time polymerase chain reaction (RT-PCR) protocols for SARS-CoV-2 detection. These target parts of the ribonucleic acid (RNA)-dependent RNA polymerase gene, the envelope (E) protein gene, or of the nucleocapsid (N) protein gene [4].

The E- and N-gene assays of Corman et al. have been integrated into our laboratory workflow at the end of January 2020. Furthermore, we have added two additional primer/probe pairs to each of the protocols. In addition to virus detection, these supplements enable the amplification of a region of the human glyceraldehyde-3-phosphate dehydrogenase (GAPDH) gene [5] and the detection of an RNA phage [6] that was added to the sample prior to the extraction of nucleic acids (NA). In any case, this assay design ensures the correct removal of the swab from the upper respiratory tract (URT) as well as the successful extraction of the NA and the reverse transcription of RNA into copy deoxyribonucleic acid (DNA) and their subsequent amplification. The viral load in the initial sample is estimated by specifying the threshold cycle (Ct) of the SARS-CoV-2 signal in the laboratory-developed triplex RT-PCR. Of note, such a method cannot quantify the virus concentration exactly [7,8] and just represents a rough estimation.

Performing RT-PCR requires a medical laboratory with well-trained staff as well as reagents and equipment for NA extraction and amplification. This is challenging in the current situation of the ongoing SARS-CoV-2 pandemic. Under the given circumstances, the time from sampling to reporting the test result can sometimes exceed 48 h [9]. Thus, easy-to-perform lateral flow assays that can be applied for rapid detection of SARS-CoV-2 antigen at the point-of-care (POCT) may be useful to deal with these limitations. However, so far only a few studies on the diagnostic performance of such tests are available [9,10,11,12,13,14].

Here, the sensitivity and specificity of a commercially available POCT for rapid detection of the SARS-CoV-2 nucleoprotein was investigated. For this, we first present data on the performance of the laboratory-developed triplex RT-PCRs, which served as a reference test in our study. Then, the defined URT samples were re-tested with the POCT and partially also in a second POCT. Finally, in order to better classify the clinical relevance of the results, we show data on the virus isolation rate in the cell culture as an expression of the viral load in the URT sample. Overall, the POCT was found to be useful for the rapid identification of highly infectious individuals.

## 2. Materials and Methods

### 2.1. Preparation of NA from URT Samples

Deep nasopharyngeal swabs were stirred in 500 µL sterile phosphate buffered saline (PBS, Lonza, Basel, Switzerland). The KingFisher Flex (Thermo Fisher Scientific, Waltham, MA USA) was preferably used for the extraction of NA from these swabs. A modified protocol for the MagMAX Viral/Pathogen kit (Thermo Fisher Scientific) which uses a reduced sample input and only two washing steps was applied. This protocol was recently published by the manufacturer as an application note to enable increased diagnostic throughput (https://www.thermofisher.com/de/de/home/life-science/dna-rna-purification-analysis/automated-purification-extraction/automated-magmax-kits-nucleic-acid-extraction/magmax-viral-pathogen-extraction-kits.html; accessed 19 November 2020) and was further adapted to our workflow. For this, a Pseudomonas phi6 phage suspension (Deutsche Sammlung von Mikroorganismen und Zellkulturen, Braunschweig, Germany) was used instead of the MS2 phage. Lysis of 150 µL sample was performed in 200 µL Binding Buffer completed with 3.8 µL Proteinase K, 3.8 µL magnetic beads and 3.8 µL phage (1:30 diluted in PBS). The first wash step was performed in 100 µL washing buffer. The second wash step was performed in 150 µL of 80 % Ethanol. Elution of NA was done in 30 µL elution buffer.

### 2.2. Triplex RT-PCR Used as Reference Test for Detection of SARS-CoV-2

The oligonucleotide and probe sequences for detection of SARS-CoV-2 N- or E-gene, human GAPDH and phi6 phage were taken from literature (Table S1), and synthesis was done at biomers.net GmbH, Ulm, Germany. The lyophylisates were dissolved in molecular-biology grade water to achieve a concentration of 100 µM in the stock solution. Primers and probes for SARS-CoV-2 E- and N-gene detection were diluted to achieve a final concentration of 16 µM and 8 µM, respectively. The primers and probe for phi6 phage were diluted to a final concentration of 10 µM and 4 µM, respectively. The primers and probe for GAPDH were diluted to a final concentration of 8 µM and 4 µM, respectively, for amplification on the ABI7500 PCR machine (ThermoFisher Scientific). If the LightCycler 2.0 (LC 2.0, Roche, Mannheim, Germany) was used for amplification, these oligonucleotides and the probe were diluted to a final concentration of 10 µM and 4 µM, respectively. The working dilutions were mixed 1:1:1 (primer:primer:probe) per target and stored at −20 °C. Out of these preparations, 1 µL of the E- or 2 µL of the N-gene mix, 1 µL of the phi6 mix, and 0.5 µL of the GAPDH mix, respectively, were added to 10 µL Luna Universal Probe One-Step Reaction Mix (2×) together with 1 µL Luna WarmStart^®^ RT Enzyme Mix (20×) and 0.5 µL Antarctic Thermolabile Uracil-DNA Glycosylase (all New England Biolabs GmbH, NEB, Frankfurt am Main, Germany) to achieve a volume of 15 µL (thus, for the E-gene triplex RT-PCR, 1 µL of water was added). PCR-plates with 96 wells (ThermoFisher Scientific) or LC 2.0 glass capillaries (Roche) were filled with 15 µL of this mastermix as well as 5 µL of the NA. After a short centrifugation step, these plates or capillaries were incubated for 10 min at room temperature and put in the ABI7500 or the LC 2.0, respectively. Cycling conditions were in accordance with the recommendations of NEB. In brief, reverse transcription (55 °C, 10 min) was followed by an initial denaturation step (95 °C, 1 min) and 45 cycles of denaturation (95 °C, 10 s) and extension (60 °C, 30 s; including reading of fluorescence). For cycling on the LC 2.0, a color compensation file had to be generated before the experiments. The number of RT-PCR cycles required for the fluorescent signal to exceed the background level (threshold) was recorded as threshold cycle (Ct) [15]. The laboratory-developed triplex RT-PCRs were validated by testing of defined External Quality Assessment (EQA) samples obtained from Instand e.V. (Düsseldorf, Germany). The mean viral load given by Instand e.V. was considered as reference. Furthermore, the N-gene triplex RT-PCR was tested against a commercial kit for simultaneous detection of N-gene, RdRp-gene and an internal control (RealAccurate^®^ Quadruplex SARS-CoV-2 PCR Kit, PathoFinder B.V., Maastricht, The Netherlands).

### 2.3. SARS-CoV-2 Antigen Tests Investigated in This Study

The performance of the NADAL^®^ COVID-19 Ag Test (Nal von Minden GmbH, Moers, Germany) was investigated in detail. For this purpose, the URT samples already present and stirred in PBS were included, considering the Ct values of the N-gene signal of the triplex RT-PCR. One-hundred microliter of the PBS/sample solution were used (20%, 100 µL/500 µL) and mixed in a tube together with three drops of the sample extraction buffer (ca. 70 µL out of the buffer bottle). After two minutes, the supplied dropper cap was attached to the tube containing the sample/buffer mix. Then this tube was inverted, and two drops were transferred to the sample field of the test cassette. The assay was visually assessed after 15 min and a photo was taken. Optical intensity of the antigen band was automatically measured with the RSS III reader (Nal von Minden).

In addition, the Panbio™ COVID-19 Antigen rapid test (Abbott GmbH, Wiesbaden, Germany) was valued. For this, 100 µL (20%) of patient sample in PBS were mixed in the supplied extraction tube together with ca. 200 µL of extraction buffer (ca. eight drops out of the buffer bottle). Then the dropping nozzle cap of the extraction tube was removed, and five drops of the sample/buffer mix were transferred to the sample field of the test cassette. The assay was visually assessed after 15 min and a photo was taken.

Twenty of the investigated SARS-CoV-2 positive original samples were re-filled with PBS and re-evaluated by RT-PCR and several POCTs (including the assays from Nal von Minden and Abbott) at the Institute of Virology, Charité Berlin, as part of another study [12].

### 2.4. Inoculation of Vero Cells with Various Concentrations of SARS-CoV-2

These experiments were conducted under biosafety level 3 conditions. Vero cells were inoculated with material from URT swabs stirred in PBS and tested as positive for SARS-CoV-2 N-gene in the triplex RT-PCR in order to correlate the virus concentrations (in means of Ct) to the capacity of viral growth. In brief, one day before inoculation, 1 × 10^5^ Vero cells (order no. 605372, CLS Cell Lines Service GmbH, Eppelheim, Germany) per well were seeded into 48-well plates (Greiner Bio-One GmbH, Frickenhausen, Germany) and incubated at 37 °C under standard conditions [16]. At the day of infection, the cells were washed with PBS and incubated at 37 °C for one hour with 50 µL of the pretested patient material and 150 µL of a cell-culture medium consisting of Dulbecco’s modified Eagle’s medium (DMEM), supplemented with 3.7 g/L NaHCO3, 4.5 g/L glucose, 2 mM L-glutamine and 1% (*v*/*v*) of the Pen-Strep-Fungi-Mix containing 10,000 U/mL penicillin, 10 mg/mL streptomycin, and 25 µg/mL amphotericin B (all reagents from Bio&SELL GmbH, Feucht, Germany). Thereafter, 200 µL of the cell-culture medium supplemented with 20% (*v*/*v*) fetal calf serum (FCS) (PAA Laboratories, Pasching, Austria) were added to a final FCS concentration of 10% (*v*/*v*). Four days after infection and incubation at 37 °C under standard conditions, 50 µL of the supernatant were used for inoculation of a new 48-well plate pre-seeded with Vero cells. Furthermore, 150 µL of the supernatant was inactivated by heat (10 min at 95 °C) and used for preparation of NA and N-gene triplex RT-PCR. After four days, supernatant was filtered through a 0.22 µM filter (Minisart^®^ Syringe Filters, Sartorius AG, Goettingen, Germany) in order to remove bacteria and fungi. Then, Vero cells, pre-seeded in the wells of a 48-well plate, were infected with 50 µL of the filtrate as described above. After four days, 150 µL of the supernatant from the second passage was tested in the same way for presence of SARS-CoV-2 N-gene while the residual supernatant was stored at −80 °C. Virus isolation was considered successful if the Ct value of the NA preparations from the supernatant was < 20. Furthermore, the cytopathic effect after two passages was investigated. For this, cells were fixed with 4% (*w*/*v*) paraformaldehyde (Sigma, St. Louis, USA) in PBS and stained with an aqueous solution of 0.1% (*w*/*v*) crystal violet (Carl Roth, Karlsruhe, Germany) and 20% (*v*/*v*) methanol (Merck, Darmstadt, Germany) [16].

## 3. Results

### 3.1. Suitability of the E- and N-Gene Specific Triplex RT-PCRs for Detection of SARS-CoV-2

The suitability of the laboratory-developed E- and N-gene triplex RT-PCRs for SARS-CoV-2 diagnostics was investigated by testing of defined EQA samples. This set also included well-characterized samples being positive for diverse endemic human respiratory coronaviruses (HCoV NL63, HCoV OC43, HCoV 229E) as well as MERS coronavirus and further respiratory viruses including parainfluenzavirus, influenza A and B virus, human metapneumovirus, respiratory syncytial virus, and rhino-/enterovirus. Both protocols were able to detect SARS-CoV-2 RNA and no cross-reactivity to the other included viruses was observed. Direct comparison of the Ct values from E- and N-gene specific triplex RT-PCRs indicated a slightly better performance of the E-gene assay (Table S2).

To determine the linearity and the detection limit under realistic conditions, the EQA samples 340059/20 (17,071,604 copies/mL) and 340064/20 (220,046 copies/mL) were diluted in a sample (respiratory swab in PBS) which was found to be free of SARS-CoV-2 RNA before. Thereby, URT samples with a defined number of SARS-CoV-2 copies/mL were generated. Then, NA from these artificial samples were tested on different LC 2.0 and ABI7500 machines and Ct values were recorded in relation to the viral load. The E- and N-gene triplex RT-PCRs differed by 2.6 Ct values. The agreement between the two assays was visually shown by the Bland-Altman plot [17] (Figure S1). An example of a run of the N-gene triplex RT-PCR is shown in Figure S2. Both laboratory-developed assays were able to reliably detect a SARS-CoV-2-positive patient sample (data not shown) for which a discrepant RT-PCR was reported due to a single mutation in the N-gene [18]. The N-gene triplex RT-PCR was also tested against a commercial RT-PCR designed for simultaneous detection of N-gene, RdRp-gene and a control. The N-gene signal differed by 2.3 Ct values. The Bland-Altman plot [17] was used to visualize the agreement between both tests (Figure S3).

### 3.2. Direct Detection of SARS-CoV-2 Antigen

**Figure 1:**
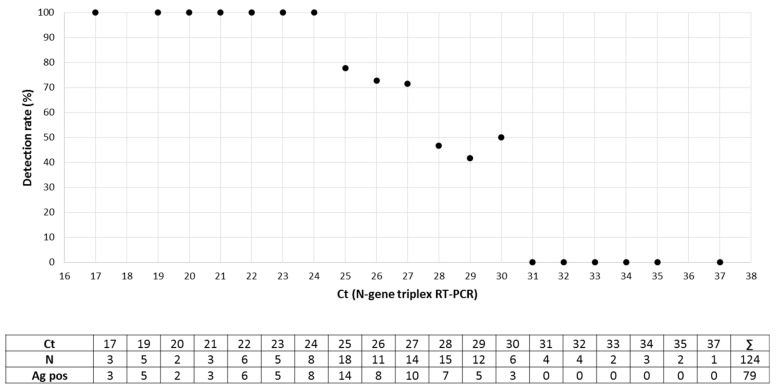
Detection of SARS-CoV-2 antigen in samples of the upper respiratory tract (*n* = 124) by the NADAL^®^ COVID-19 Ag test (Nal von Minden). The detection rate was assessed in terms of the threshold cycle (Ct) of the N-gene triplex RT-PCR. The raw data are listed in the table below the figure.

**Figure 2:**
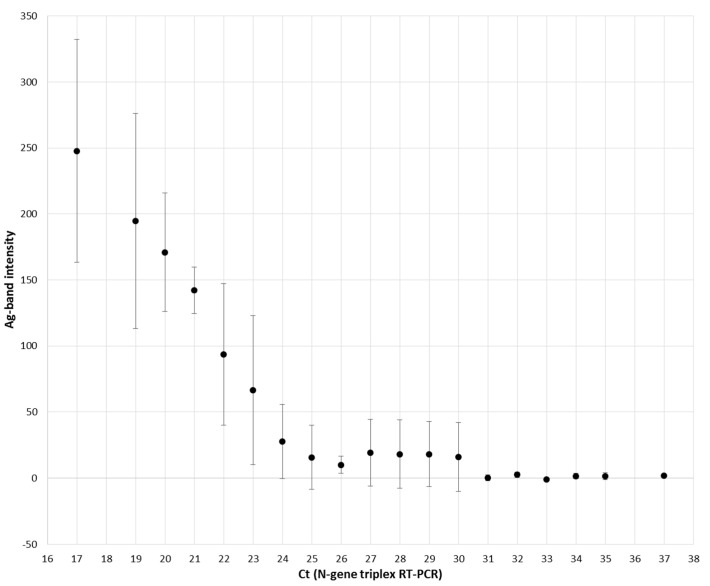
Optical intensity of the SARS-CoV-2 antigen band observed in the NADAL^®^ COVID-19 Ag test (Nal von Minden). The intensity was measured automatically by scanning and rated in relation to the threshold cycle (Ct) of the N-gene triplex RT-PCR. Mean intensities and standard deviations are given. The samples correspond to those in Figure 1.

A total of 134 URT samples were re-tested with the NADAL^®^ COVID-19 Ag test. All samples were pre-characterized by a positive (124 samples, Ct of 17 to 37) or a negative (10 samples) result in the N-gene triplex RT-PCR. For re-testing, 100 µL out of 500 µL were used and mixed with ca. 70 µL of the extraction buffer, and two drops were transferred to the sample field. The antigen test had a sensitivity of 100% (32/32) for samples with a high viral concentration (Ct < 25). For lower viral loads with a Ct of 25 to 30, the positive detection rate varied from 77.8% (Ct 25, 14/18) to 41.7% (Ct 29, 5/12). Thus, overall sensitivity for Ct ≤ 30 was found to be 73.1% (79/108). Samples with rather low viral loads (Ct > 30, 16/16) were always tested negative in the antigen test (Figure 1). The intensity of the antigen test band was measured by automatic scanning. A decrease in mean intensities was found between Ct 17 and Ct 24. From Ct 25 to Ct 30 mean intensities reached a stable low-level plateau. Thereafter, the baseline was reached (Figure 2). Exemplary tests of ten SARS-CoV-2-negative samples also showed negative results in the antigen test. Detailed investigations into the specificity of the commercial POCT were not carried out because extensive data from the manufacturer were available in the manual. In addition, this assay showed a specificity of 99.3% in another study [12].

**Figure 3:**
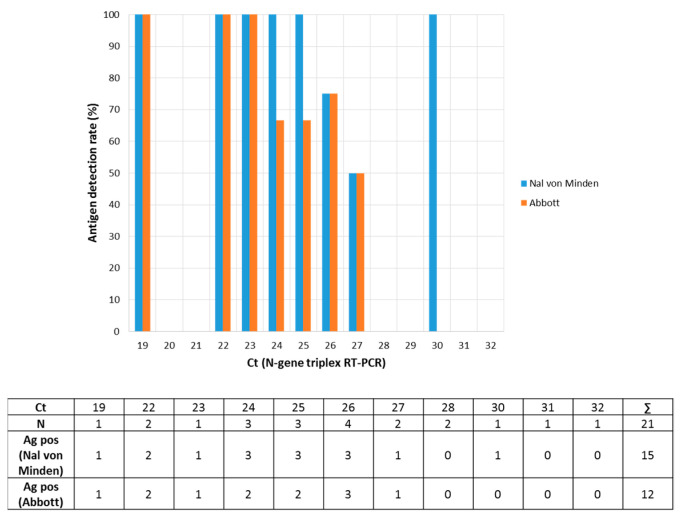
Head-to-head comparison of two POCTs for detection of SARS-CoV-2 antigen. Upper respiratory tract samples which have been already tested in the NADAL^®^ COVID-19 Ag test (Nal von Minden) were re-tested with the Panbio™ COVID-19 Antigen rapid test (Abbott, *n* = 21). The raw data are listed in the table below the figure.

For comparison, the Panbio™ COVID-19 Antigen rapid test was valued by the head-to-head testing of 21 samples from Ct 19 to Ct 32. For the Panbio™ assay, 100 µL of the sample in PBS were mixed with ca. 200 µL of extraction buffer, and five drops of this solution were transferred to the sample field. The qualitative results were similar (Figure 3, Table 1; Fisher-Test, two-tailed *p* value equals 0.5204; https://www.graphpad.com/quickcalcs/contingency2/, accessed 19 November 2020).

### 3.3. Virus Growth Rate in Relation to Viral Load

**Figure 4:**
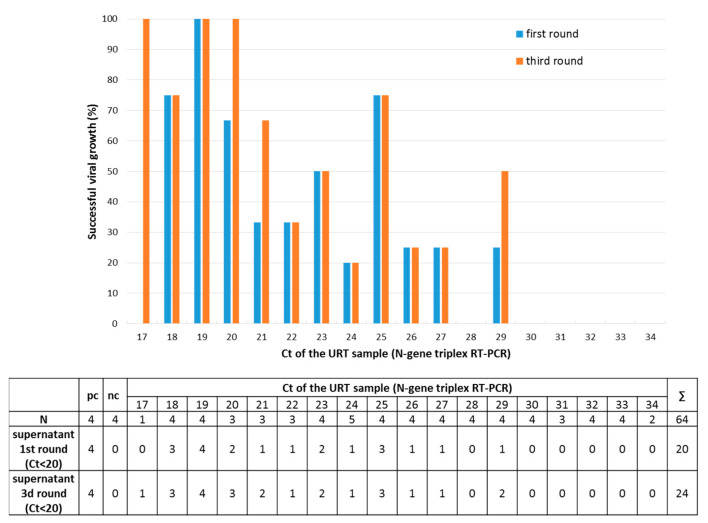
Isolation of SARS-CoV-2 from upper respiratory tract (URT) samples in Vero cells. A successful isolation in cell culture was assumed if the supernatant of the corresponding well had a threshold cycle (Ct) < 20 in the N-gene triplex RT-PCR. The isolation rate was estimated as a function of viral load by mean of the Ct (N-gene triplex RT-PCR) of the original URT sample. The results after the first round on Vero cells and after the third round are shown. Four positive and negative controls (pc, nc) were included. The table below the figure lists the result per URT sample and the corresponding Ct. The cytopathic effect observed after the third round in Vero cells is documented in Figure 5.

**Figure 5:**
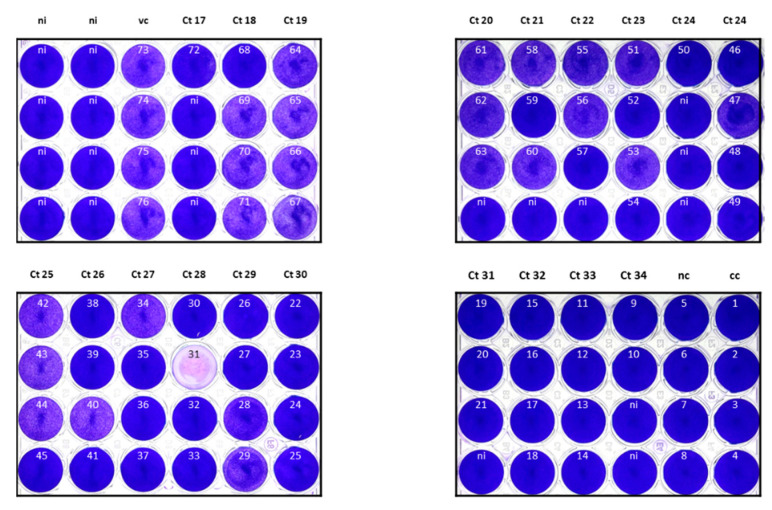
Cytopathic effect after two passages (third round) of 64 upper respiratory tract samples (no. 9–72) in Vero cells with respect to the threshold cycle (Ct) values of the original sample (N-gene triplex RT-PCR). For comparison, positive controls (vc, no. 73–76; own SARS-CoV-2 isolates), negative controls (nc, no. 5–8; SARS-CoV-2 free patient samples) and cell controls (cc, no. 1–4, and ni; cells in medium) are included. Note that the destroyed cell layer in no. 31 represents an artifact and does not correlate to the result of N-gene triplex RT-PCR from the supernatant (compare Figure 4).

The ability to grow a virus in Vero cell culture was considered a surrogate marker for the patient’s infectivity. For this investigation, Vero cells pre-seeded in 48-well plates were inoculated with 50 µL (10%) of the PBS solution obtained from 64 URT samples pretested by N-gene triplex RT-PCR with different SARS-CoV-2 loads ranging from Ct 17 to Ct 34. Four days after inoculation, NAs were extracted from 150 µL of the supernatant and tested in the N-gene triplex RT-PCR. After this first round, 31.3% (20/64) of samples yielded a Ct < 20. For these samples, it was assumed that the virus culture was successful. After the following two passages (third round), virus isolation was successful in four additional samples (Ct < 20, 37.5%, 24/64). No SARS-CoV-2 could be isolated if the original URT samples had a Ct > 29 (Figure 4). The seeded cells were also fixed, stained with crystal violet and photo documented. The supernatants of wells in which a destroyed cell layer can be seen also have a Ct < 20 in the N-gene triplex RT-PCR, which corresponds to a successful isolation of SARS-CoV-2 (Figure 5).

## 4. Discussion

The real-time reverse transcription PCR represents the gold standard for SARS-CoV-2 detection. Several PCR protocols have been released by the World Health Organization, the Centers for Disease Control of the United States, and various other health authorities [19]. Here, we make use of the oligonucleotide and probe sequences suggested by Corman and colleagues for detection of the E- and N-gene [4]. These were supplemented by primers and probes for the parallel detection of GAPDH (to demonstrate correct sampling) and an RNA phage (to ensure correct extraction of RNA and successful transcription into DNA followed by successful PCR). Both triplex RT-PCR protocols are suitable for SARS-CoV-2 diagnostics but slightly differ in their performance. In this study, the E-gene assay was found to show SARS-CoV-2 specific signals 2.6 Ct values before the N-gene assay. The agreement of the laboratory-developed N-gene triplex RT-PCR with a commercially available RT-PCR is good. The robust in-house assay reliably detects a SARS-CoV-2 infection and is less prone to the risk of possible contamination due to the use of uracil DNA glycosylase. This test may be further optimized to achieve the performance of the E-gene variant. Slight differences in SARS-CoV-2 RT-PCR performance have also been observed by others [19]. Conducting PCR assays requires trained laboratory personnel and a variety of equipment and reagents to process and test the samples. The device and reagent resources are a limiting factor in the current phase of the pandemic. There is therefore an urgent need for simple tests that can be carried out independently of medical laboratories and that can quickly and reliably detect a SARS-CoV-2 infection. Immunochromatographic cassette tests for the detection of viral antigens by lateral flow technique at the point-of-care could solve this problem. Such assays have recently been developed and brought to market by several manufacturers. However, so far there have been few independent studies evaluating their performance [9,10,11,12,13,14].

Here, a POCT for detection of SARS-CoV-2 nucleoprotein was evaluated and compared to our N-gene specific laboratory-developed triplex RT-PCR as well as to another POCT. The NADAL^®^ COVID-19 Ag test was able to reliably detect URT samples with a high viral load (Ct < 25; 100%, 32/32). For samples with a medium viral load (Ct 25 to 30), the sensitivity decreased (61.8%, 47/76), while all samples with a low viral load were tested negative by the POCT. For highly positive samples (Ct < 25), a decreasing intensity of the antigen band was measured when the viral load decreased. So far this has only been reported qualitatively as band differences visible to the naked eye [9]. Considering the relatively small sample size (*n* = 21), the second POCT examined in our study had an identical diagnostic performance. It can be assumed that the sensitivity of both antigen tests was underestimated and is even higher because the URT swabs were already stirred into PBS and pretested in the triplex RT-PCR several hours before. Furthermore, not the entire amount of each sample was used for testing. In this regard, our study differs somewhat from the setting at the point-of-care and the recommendations of the manufacturers. This may partly explain the discrepancy in sensitivity for samples in the range from Ct 20 to 30 given by Nal von Minden (97.6%) and determined by us (71%, 71/100). It should be kept in mind that Ct values differ between RT-PCR tests (as also observed in our study). The Ct value can therefore only provide an orientation in order to estimate the virus load in categories like “high positive”, “medium” and “low positive”. The excellent detection of high positive samples by several SARS-CoV-2 antigen assays including POCTs has been also reported by others [9,10,11,12,13,14,20,21]. An evaluation of all positive SARS-CoV-2-PCRs (including follow-up examinations) that we had between 30 August 2020 and 30 October 2020, showed that 66.8% (508/761) of the samples had Ct values > 24 and 25.4% of samples exhibited Ct values > 30 (193/761) (Figure S4). The diagnosis of a SARS-CoV-2 infection would therefore be too uncertain for a medical laboratory based on the antigen detection alone. However, it is known that patients have a high viral load in their URT in the first days of infection [22,23,24,25,26,27,28]. Thus, POCT for the detection of SARS-CoV-2 antigen may reliably identify patients in this period who can then be isolated to prevent further spreading of infection [9,10,11,12,14,29]. The small diagnostic window in the very early phase of the infection, in which the RT-PCR can already detect the infection, but the antigen level is not high enough for the POCT, can easily be closed by repeated testing [29]. Another important point is the identification of patients who are no longer infectious (but still positive in the RT-PCR) and can be safely released from isolation [12,29]. The reliable identification of infectious patients by the investigated POCT is demonstrated by our cell-culture experiments. If we consider the growth of SARS-CoV-2 in Vero cells as a function of the viral load in the URT sample and take this as a surrogate for infectivity of the patient, we can clearly show that this rate decreases with decreasing viral load. Accordingly, we were not able to isolate SARS-CoV-2 in Vero cells from URT samples with a Ct > 29 in the N-gene triplex RT-PCR. Such samples will be tested antigen negative in the POCT. The results of our cell-culture experiments are comparable to a preprint on the clinical evaluation of the Roche/SD Biosensor rapid antigen test [9] and will also allow us a better interpretation of the own Ct values. A recent review emphasizes that virus growth rate correlates with viral load. In addition, the authors report that with a few exceptions, cultivation is only successful within the first nine days after the onset of the disease, even if some patients have a high viral load in the RT-PCR for weeks [28]. This could explain why no virus could be isolated in some of the highly positive samples in our study, especially since we have no information on the onset and kind of symptoms.

Both POCTs examined here are characterized by their user-friendliness and the relatively low costs of well under 10 euros per patient sample. By scanning the intensity of the antigen band, the evaluation of the test can be simplified and standardized. As for all assays, the diagnostic value of the POCT also depends on the pretest probability by means of disease prevalence and the underlying test setting (https://www.cdc.gov/coronavirus/2019-ncov/lab/resources/antigen-tests-guidelines.html, accessed 19 November 2020). Therefore, when the prevalence is low, the risk of false positives is significantly higher. As such, positive POCT results should be confirmed by RT-PCR [11].

## 5. Conclusions

Overall, in the current situation of the pandemic with the increasing prevalence of SARS-CoV-2 infections, POCT can be a useful tool for the rapid identification of individuals in their first phase of infection. In addition, these tests can help identify patients who can no longer transmit the virus.

## Figures and Tables

**Table 1:** Head-to-head comparison of two POCTs for detection of SARS-CoV-2 antigen.

		Nal Von Minden (NADAL^®^ COVID-19 Ag Test)		
		pos	neg	*n*
**Abbott** **(Panbio™ COVID-19 Antigen rapid test)**	**pos**	12	0	12
	**neg**	3	6	9
	***n***	15	6	21

## Supplementary Materials

The following are available online at https://www.mdpi.com/2076-2607/9/1/58/s1, Figure S1: Linearity (A) and agreement (B) of the SARS-CoV-2 signal in the E- and N-gene triplex RT-PCRs, Figure S2: Example of a N-gene triplex RT-PCR experiment on the ABI7500, Figure S3: Comparison of the N-gene triplex RT-PCR to a commercial SARS-CoV-2 RT-PCR, Figure S4: Frequency of SARS-CoV-2 RT-PCR threshold cycle (Ct) values in infected individuals, Table S1: Primer and probe sequences of the laboratory-developed triplex RT-PCRs for detection of SARS-CoV-2, phi 6 phage and GAPDH, Table S2: Specific detection of SARS-CoV-2 RNA by the laboratory-developed triplex RT-PCRs.
