# Supplementary material for: Performance of a Point-of-Care Test for the Rapid Detection of SARS-CoV-2 Antigen
Source: Microorganisms. 2020 Dec 28;9(1):58. doi: 10.3390/microorganisms9010058 (PMC7823488; doi:10.3390/microorganisms9010058)
Supplement: Supplementary file 1 [file microorganisms-09-00058-s001.zip › supplementary_material/Supplementary_Figure_1.pdf]

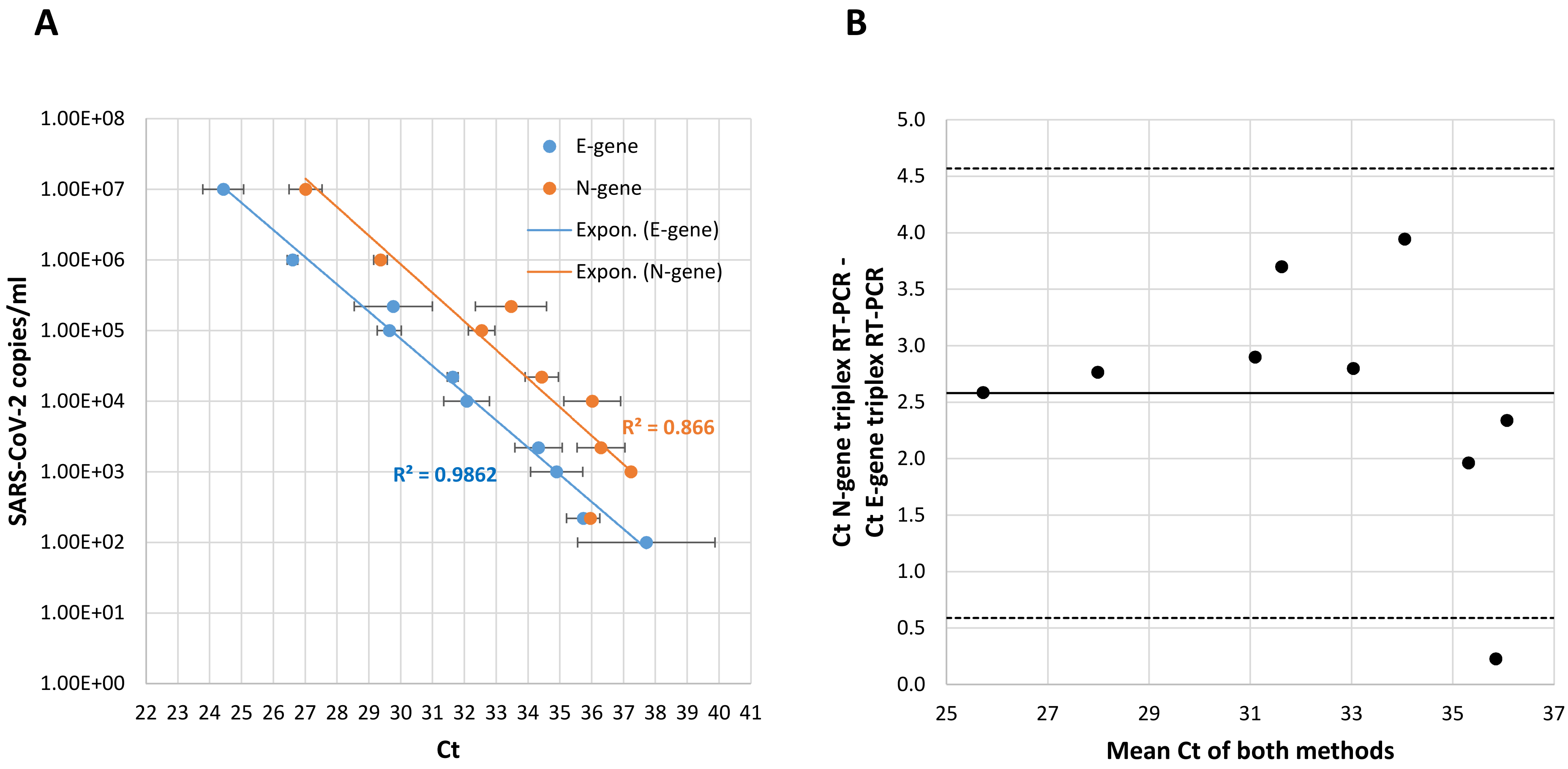

Figure S1: Linearity (A) and agreement (B) of the SARS-CoV-2 signal in the E- and N-gene triplex RT-PCRs. Two EQA-samples with pre-known SARS-CoV-2 load were diluted in a SARS-CoV-2 free human respiratory sample to obtain clinical specimens with defined SARS-CoV-2 copies/ml. Mean threshold cycle (Ct) values (and standard deviations) from up to six runs are given (A). The Bland-Altman-Plot (B) shows the bias (mean difference between the Ct values of both methods, solid line) with the 95% limits of agreement (dashed lines).
