# Supplementary material for: Performance of a Point-of-Care Test for the Rapid Detection of SARS-CoV-2 Antigen
Source: Microorganisms. 2020 Dec 28;9(1):58. doi: 10.3390/microorganisms9010058 (PMC7823488; doi:10.3390/microorganisms9010058)
Supplement: Supplementary file 1 [file microorganisms-09-00058-s001.zip › supplementary_material/Supplementary_Figure_2.pdf]

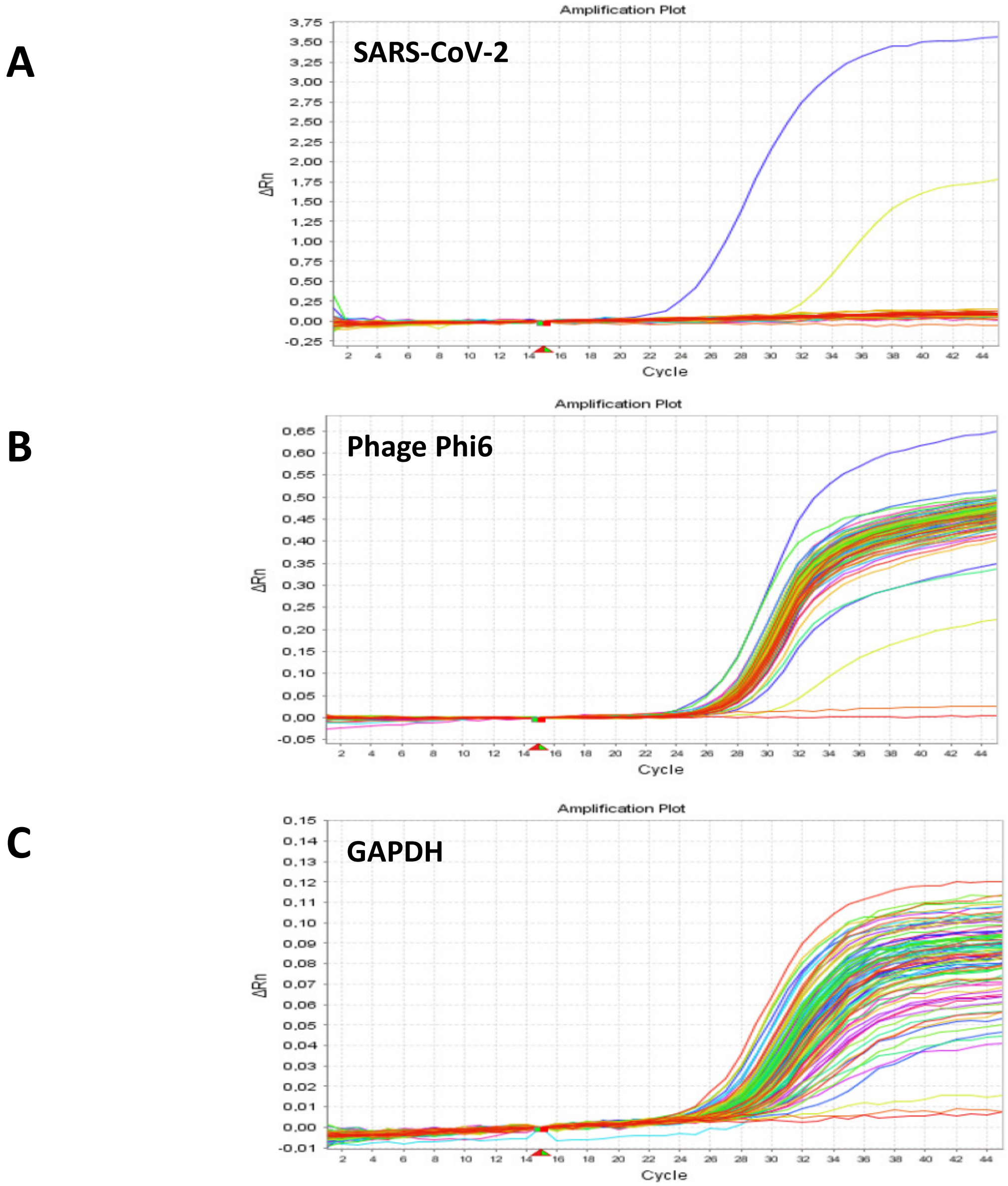

Figure S2: Example of a N-gene triplex RT-PCR experiment on the ABI7500. Samples from the upper respiratory tract (URT, N=93) and a combined SARS-CoV-2/phage Phi6 positive control (light green, parts A and B; GAPDH negative, part C) as well as two negative controls (red/orange in A-C) were examined. A SARS-CoV-2 infection was detected in one patient (blue, part A). The mean threshold cycle (Ct) of the RNA phage signal in the spiked URT samples is 26.9 (standard deviation, SD=0.5) and that of the GAPDH signal is 28.1 (SD=1.4).

2020-11-27-03-P01
