# Supplementary material for: Performance of a Point-of-Care Test for the Rapid Detection of SARS-CoV-2 Antigen
Source: Microorganisms. 2020 Dec 28;9(1):58. doi: 10.3390/microorganisms9010058 (PMC7823488; doi:10.3390/microorganisms9010058)
Supplement: Supplementary file 1 [file microorganisms-09-00058-s001.zip › supplementary_material/Supplementary_Figure_3.pdf]

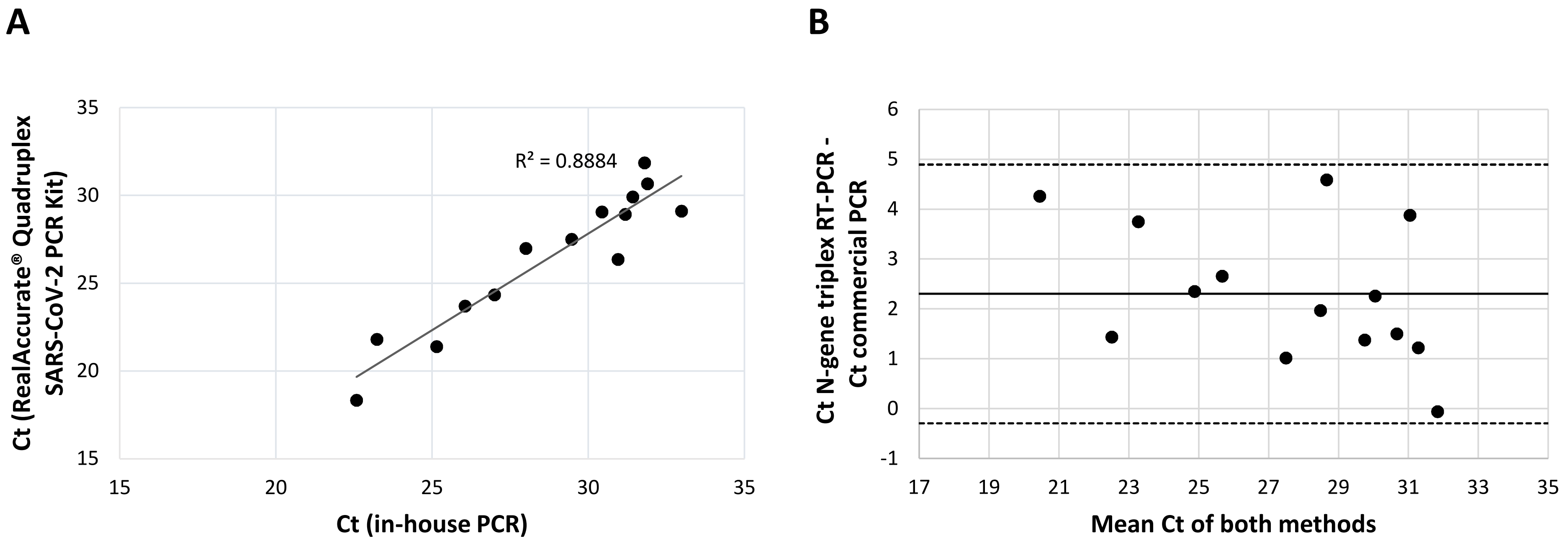

Figure S3: Comparison of the N-gene triplex RT-PCR to a commercial SARS-CoV-2 RT-PCR. Fourteen SARS-CoV-2 positive samples were tested in parallel by both assays and the threshold cycle (Ct) of the N-gene signals were compared (A). The Bland-Altman-Plot (B) shows the bias (mean difference between the Ct values of both methods, solid line) with the 95% limits of agreement (dashed lines).
