# Supplementary material for: Performance of a Point-of-Care Test for the Rapid Detection of SARS-CoV-2 Antigen
Source: Microorganisms. 2020 Dec 28;9(1):58. doi: 10.3390/microorganisms9010058 (PMC7823488; doi:10.3390/microorganisms9010058)
Supplement: Supplementary file 1 [file microorganisms-09-00058-s001.zip › supplementary_material/Supplementary_Figure_4.pdf]

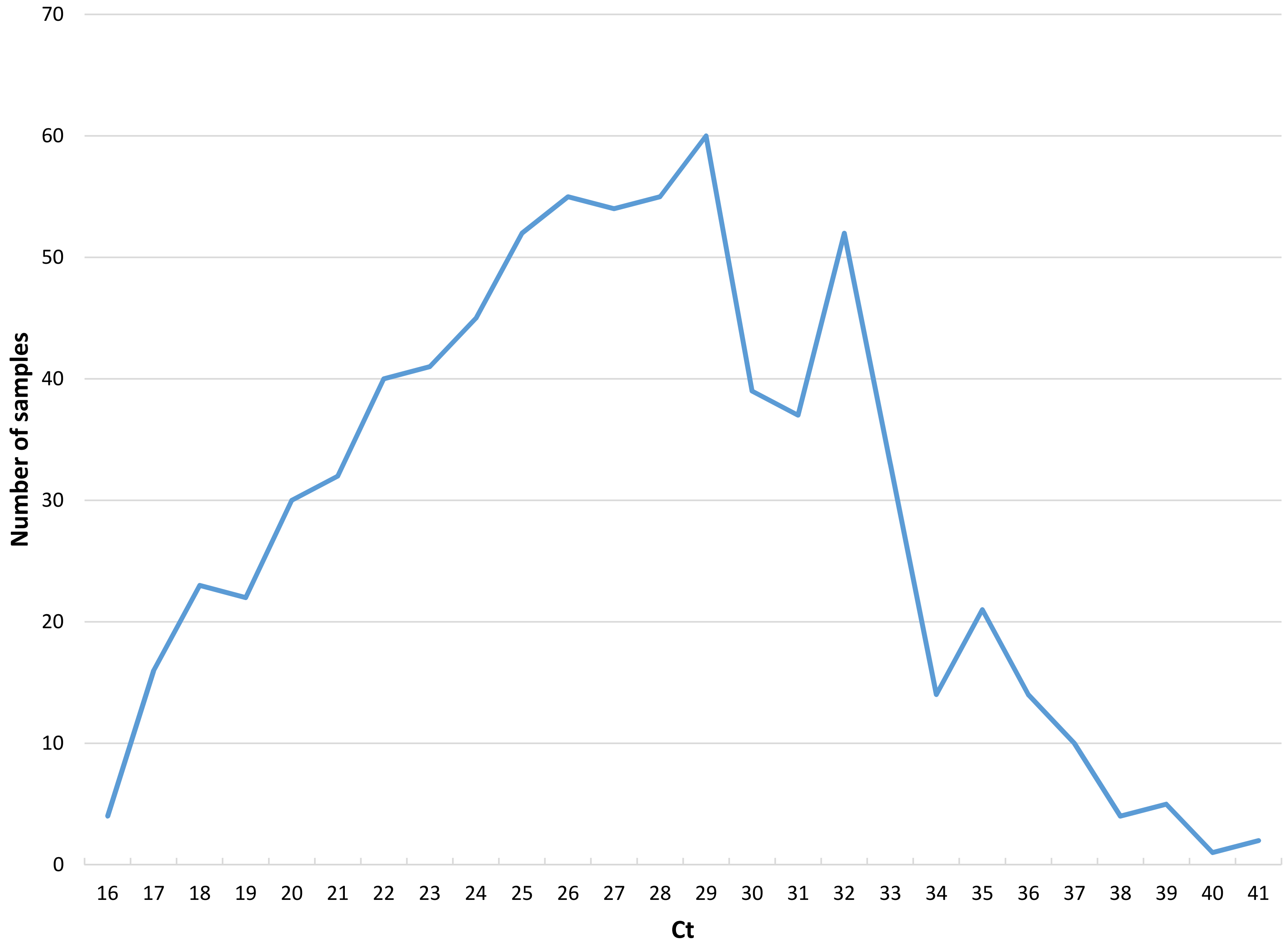

Figure S4: Frequency of SARS-CoV-2 RT-PCR threshold cycle (Ct) values in infected individuals. Results of cumulative data from our diagnostic laboratory, 30 August 2020 to 30 October 2020.
