# Supplementary material for: Performance of a Point-of-Care Test for the Rapid Detection of SARS-CoV-2 Antigen
Source: Microorganisms. 2020 Dec 28;9(1):58. doi: 10.3390/microorganisms9010058 (PMC7823488; doi:10.3390/microorganisms9010058)
Supplement: Supplementary file 1 [file microorganisms-09-00058-s001.zip › supplementary_material/Table S1.pdf]

Table S1: Primer and probe sequences of the laboratory-developed triplex RT-PCRs for detection of SARS-CoV-2, phi 6 phage and GAPDH

**Target**	**Designation of forward (F) and reverse (R) primer and probe (P)**	**Sequence (5′-3′)**<br>**5′-dye and 3′-quencher (probe)**	**Reference**
SARS-CoV-2 *E-gene*	SARS2-E-F*	ACAGGTACGTTAATAGTTAATAGCGT	[4]
	SARS2-E-R*	ATATTGCAGCAGTACGCACACA	
	SARS2-E-P*	FAM-ACACTAGCCATCCTTACTGCGCTTCG-BMNQ530	
SARS-CoV-2 *N-gene*	SARS2-N-F*	CACATTGGCACCCGCAATC	
	SARS2-N-R*	GAGGAACGAGAAGAGGCTTG	
	SARS2-N-P*	FAM-ACTTCCTCAAGGAACAACATTGCCA-BMNQ530	
Pseudomonas Phi 6 Phage	Phi6-F	TGGCGGCGGTCAAGAGC	[6]
	Phi6-R	GGATGATTCTCCAGAAGCTGCTG	
	Phi6-P	HEX-CGGTCGTCGCAGGTCTGACACTCGC-BMNQ535	
GAPDH	GAPDH-F	GGACTGAGGCTCCCACCTTT	[5]
	GAPDH-R	GCATGGACTGTGGTCTGCAA	
	GAPDH-P (LC 2.0)	Atto490LS-ATCCAAGACTGGCTCCTCCCTGC-BMNQ620	
	GAPDH-P (ABI 7500)	Cy5-ATCCAAGACTGGCTCCTCCCTGC-BHQ2	
*will also detect the SARS-CoV from 2002/2003 and SARS-related CoVs from bats (see [4])			
