# Supplementary material for: Performance of a Point-of-Care Test for the Rapid Detection of SARS-CoV-2 Antigen
Source: Microorganisms. 2020 Dec 28;9(1):58. doi: 10.3390/microorganisms9010058 (PMC7823488; doi:10.3390/microorganisms9010058)
Supplement: Supplementary file 1 [file microorganisms-09-00058-s001.zip › supplementary_material/Table S2.pdf]

Table S2: Specific detection of SARS-CoV-2 RNA by the laboratory-developed triplex RT-PCRs

Virus	EQA (Instand e.V.)	SARS-CoV-2 copies/ml (Instand e.V.)	Ct-value	
			E-gene	N-gene
Coronaviruses				
SARS-CoV-2	340059/20	17,071,604	22.80	25.66
SARS-CoV-2	340063/20	2,198,982	25.59	28.71
SARS-CoV-2	340069/20	268,471	28.51	31.78
SARS-CoV-2	340064/20	220,046	28.82	32.37
SARS-CoV-2	340061/20	25,978	32.44	36.24
SARS-CoV-2	340073/20	25,002	32.58	35.73
SARS-CoV-2	340066/20	2943	35.58	38.25
SARS-CoV-2	340071/20	294	38.10	neg
MERS-CoV	340051/19		neg	neg
MERS-CoV	340067/20		neg	neg
HCoV 229E	340052/19		neg	neg
HCoV 229E	340065/20		neg	neg
HCoV 229E	340068/20		neg	neg
HCoV NL63	340047/19		neg	neg
HCoV NL63	340072/20		neg	neg
HCoV OC43	340050/19		neg	neg
HCoV OC43	340060/20		neg	neg
HCoV OC43	340074/20		neg	neg
Coronavirus negative	340062/20		neg	neg
Coronavirus negative	340070/20		neg	neg
Other respiratory viruses				
Enterovirus (Coxsackievirus A21)	372077/19		neg	neg
Influenza A virus (A(H1N1)pdm09)	370120/20		neg	neg
Influenza B virus (B/Victoria-Linie)	370119/20		neg	neg
Metapneumovirus (Type A)	385047/19		neg	neg
Parainfluenzavirus (PIV-2)	388052/20		neg	neg
Respiratory syncytial virus (RSV A)	359057/19		neg	neg
Rhinovirus (HRV A Type 30)	393035/19		neg	neg
